# Biomarkers of aging in real life: three questions on aging and the comprehensive geriatric assessment

**DOI:** 10.1007/s11357-022-00613-4

**Published:** 2022-07-07

**Authors:** Marta Zampino, M. Cristina Polidori, Luigi Ferrucci, Desmond O’Neill, Alberto Pilotto, Manfred Gogol, Laurence Rubenstein

**Affiliations:** 1 Longitudinal Studies Section, Translational Gerontology Branch, National Institute on Aging, National Institutes of Health, Baltimore, MD USA; 2 Aging Clinical Research, Department II of Internal Medicine and Center for Molecular Medicine Cologne, Faculty of Medicine and University Hospital Cologne, University of Cologne, Cologne, Germany; 3 Cologne Excellence Cluster On Cellular Stress- Responses in Aging-Associated Diseases (CECAD), Faculty of Medicine and University Hospital Cologne, University of Cologne, Cologne, Germany; 4 Tallaght University Hospital and Trinity College Dublin, Tallaght University Hospital, Trinity Centre for Health Sciences, Dublin, Ireland; 5 Geriatrics Unit, Department of Geriatric Care, Orthogeriatrics and Rehabilitation, Galliera Hospital, Genoa, Italy; 6 Department of Interdisciplinary Medicine, University of Bari, Bari, Italy; 7 Trauma Department, Orthogeriatric Unit, Hannover Medical School, Hannover, Germany; 8 Institute of Gerontology, University of Heidelberg, Heidelberg, Germany; 9 Department of Geriatric Medicine, University of Oklahoma, Oklahoma City, OK USA

**Keywords:** Biological aging, Comprehensive geriatric assessment, Multidimensional prognostic index, Frailty

## Abstract

Measuring intrinsic, biological age is a central question in medicine, which scientists have been trying to answer for decades. Age manifests itself differently in different individuals, and chronological age often does not reflect such heterogeneity of health and function. We discuss here the value of measuring age and aging using the comprehensive geriatric assessment (CGA), cornerstone of geriatric medicine, and operationalized assessment tools for prognosis. Specifically, we review the benefits of employing the multidimensional prognostic index (MPI), which collects information about eight domains relevant for the global assessment of the older person (functional and cognitive status, nutrition, mobility and risk of pressure sores, multi-morbidity, polypharmacy, and co-habitation), in the evaluation of the functional status, and in the prediction of health outcomes for older adults. Further integration of biological markers of aging into multidimensional prognostic tools is warranted, as well as actions which could facilitate prognostic assessments for older persons in all healthcare settings.

## Can we measure what we struggle to define?

There is no uniform consensus definition of age and aging. The Medical Subject Heading definition in Medline reads: “The gradual irreversible changes in structure and function of an organism that occur as a result of the passage of time,” but such definition has serious flaws because it fails to include the broader context of plasticity, growth, and development during the life course.

The proportion of adults aged 65 and older is expanding worldwide due to a dramatic increase in life expectancy that occurred over the past century, and a clear definition of aging is becoming increasingly relevant. The gain in longevity has not been accompanied by a parallel lengthening of the period of life that is free from illnesses, since the current disease treatments too often decrease mortality without preventing or reversing the decline in overall health.

In the past, most of aging research was rooted in the hypothesis that diseases and aging are two distinct processes [1]. However, as our understanding of the biological mechanisms that cause disease has improved, scientists realized that a clear distinction between aging and disease was only possible at the clinical level, as most of the biological changes that occur with aging were also found to play a role in the development of chronic diseases. At the same time, progress in the biology of aging suggested that the pace of intrinsic aging, the strongest risk factor for specific chronic diseases and for multimorbidity, may be modifiable.

Multimorbidity, the condition of being affected by multiple chronic diseases, is a frequent clinical condition in persons 65 and older. However, there is evidence that the co-occurrence of chronic diseases in the older population follows a pattern that is not consistent with pure chance, and some individuals appear to “attract diseases” more than others, while others are unusually resistant [2]. Under the assumption that the pace of intrinsic aging at the biological level is relevant to the susceptibility to multiple diseases, it has been hypothesized that the pace of aging is highly heterogeneous across individuals. Over the past years, there has been intensive research on how to measure the pace of aging and identify individuals that “age faster than others” and therefore have higher risk of accelerated multimorbidity, disability, and frailty [3].

At the extreme of the severity spectrum of accelerated aging is the status of frailty. The importance of frailty as a clinical entity is now widely recognized by the medical community, although the criteria for its definition are still a matter of discussion. According to the definition proposed by Fried and collaborators, frailty is a syndrome characterized by muscle weakness, slowed gait, low physical activity, perceived low energy, and unintentional weight loss, not otherwise explained by a distinct disease pathogenesis [4].

Each one of these signs and symptoms may predict the development of the full syndrome, with weakness as the most common early predictor [5]. An alternative definition, which has been also widely used in the literature and in geriatric medicine, is the frailty index of accumulative deficit, which considers the accumulation of 30 or more co-morbidities, symptoms, diseases, disabilities, or any deficiencies, and expresses the degree of frailty as the fraction of the pre-defined impairments detected in a specific individual [6]. Consistent with the idea that frailty represents the ultimate consequence of accelerated aging, the prevalence of this condition increases geometrically with aging and predicts multiple adverse health outcomes, such as disability, loss of independence, hospitalization, and mortality [4, 6, 7]. Moreover, the presence of frailty syndrome is strongly correlated with cognitive decline and the development of most “geriatric syndromes,” clinical conditions that do not fit into nosological disease categories but have deep impact on functionality and quality of life in older persons.

However, measuring accelerated aging is substantially more difficult in the pre-clinical phase, when older persons are still cognitively and functionally intact and are not affected by overt multimorbidity. Attempts to measure “aging” are widespread in the literature of the last 20 years, but they have become more frequent and conceptually explicit over the past few years. A number of studies have operationalized the pace of aging by combining information on multiple phenotypes that typically emerge over the aging process. Examples are the “allostatic load” by Seeman and collaborators and the indexes proposed by Levine and Belsky [3, 8, 9]. Although the authors of these metrics have used the term “biological aging,” they are not capturing a biological dimension. Furthermore, each of these metrics is relevant to a different facet of the aging process or relate to different definitions of aging. Nonetheless, if the validity of these metrics could be robustly demonstrated, they could become precious tools in clinical applications. For example, the identification of “accelerated aging” in a pre-clinical state may trigger special diagnostic and intervention strategies; they could be used to track the effectiveness of interventions that supposedly reduce the rate of aging, or they may be used to predict the probability that certain individuals develop complications after an aggressive medical intervention.

The development of “true” biological metrics of aging is also an active area of investigation and several “aging clocks” have been developed from gene-expression, DNA-methylation, and proteomic data. Theoretically, these indexes should capture the biological mechanism of aging and may reveal trajectories of aging that are different between individuals. Until very recently, these tools have been tuned on chronological age and therefore have limited capacity to identify individuals who deviate from it toward accelerated health deterioration. Indeed, the predictive validity of these tools is limited. A new generation of “clocks” is emerging in the literature tuned on health characteristics, and potentially more clinically useful [10].

**Fig. 1:**
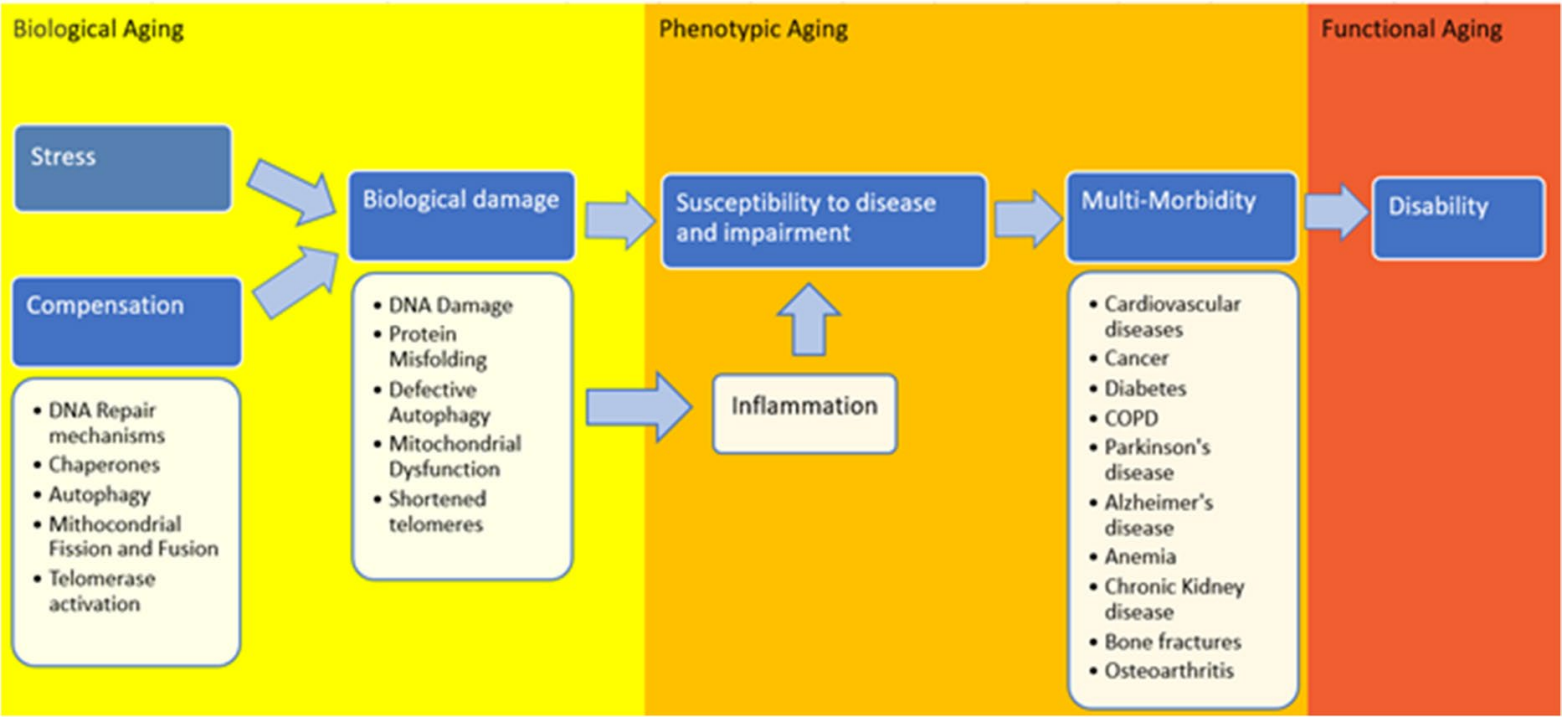
Biological, phenotypic, and functional aging and examples of the mechanisms involved

Also, beyond the obvious clinical application, this research may help identifying the biological mechanisms underneath accelerated aging and the development of chronic diseases and in particular the mechanisms of resilience. The concept of resilience is key to the study of aging. Aging can be conceptualized as a continuous, dynamic interplay between damage accumulation and resilience strategies that repair the damage and reestablish the homeostatic conditions. The ability of the organism to face the stressors with the appropriate homeostatic fluctuations is at the heart of its fitness, and it seems to get progressively weaker over the course of a lifespan. The word resilience comes from the Latin term *resiliens*, present participle form of “*resilire*: to spring back, rebound” and seems perfectly suitable to describe this capability. A highly resilient individual will be able to fully recover after major acute illness and, on the contrary, a poorly resilient individual undergoes decompensation even for a minimal stress, such as a cold. Between these two extremes lays a wide range of conditions. It is likely that the rate of biological aging would be more related to resilience mechanism rather than damage accumulation. Unfortunately, while intuitively sound, the concept of resilience is difficult to operationalize in clinical terms. The putative biological mechanisms occurring at a cellular level include mitochondrial dysfunction, increased oxidative stress, DNA damage and telomere shortening, changes in DNA methylation, deregulated nutrient-sensing, and stem cell exhaustion [11]. On a more systemic level, hormonal dysregulation, chronic inflammatory state and adaptive immune system decline, changes in body composition, muscle wasting and fat infiltration, energy unbalance, weight loss, and neurodegeneration are among the events that may be implicated in the process of aging (Fig. 1) [12–19]. However, it is important to keep in mind that the concepts of aging, damage, and resilience are still matter of debate.

In medicine, the tight exchange between basic science and clinical overtness is key to successful innovation, and the medicine of the aged person is no exception. Following the longstanding effort of the geriatric medicine towards reducing the gap between the science of aging and the care for the older chronic ill, multimorbid, functionally impaired person, Geroscience offers a solid attempt to overcome the “know-do-gap”[20] and move towards a meaningful, bidirectional trading of evidence able to inform solid research and thrive biomedical discoveries to maintain healthy and active aging.

The major obstacle to the identification of groundbreaking solutions bridging aging research and geriatrics is the definition of a common, age-attuned language in the scientific community. As displayed in Table 1, several terms used in the field of gerontology and geriatrics currently undergo, and are in need of, adjustments, as our knowledge of common mechanisms of aging and of the older generation expands [21–23]. There is an urgent need for harmonizing and structuring the terminology related to aging in medicine and in gerontology; otherwise, the research in the field will not be comparable and evidence not applicable. As feed-back, feed-forward, and content exchange between aging medicine and aging research is essential to inform both research outlooks and clinical interventions, the potential consequences of semantic confusion are disastrous and will mainly concern the inadequate care of a steadily increasing number of older persons worldwide. A strong communication between the two outlooks seems crucial for the ultimate goal of caring for the elderly.

**Table 1:** Terminology related to aging medicine in human beings

Aging	The human condition of becoming old
Disability	The International Classification of Functioning, Disability and Health (ICF) defines disability as a superfamily of impairments, activity limitations, and participation restrictions. Disability is the interaction between individuals with a health condition and personal and environmental factors (WHO 2006)
	Years lived with disability = YLD
Frailty	State of decreased reserve capacity and increased vulnerability to stressors
Geriatrics	The discipline dealing with the medical, mental, functional, and social aspects of older persons
Gerontology	Umbrella term for scientific disciplines studying the aging process
Healthy life expectancy	Disability-free life expectancy = DFLE
Life expectancy	The average time an organism is expected to live
Lifespan	The maximal duration of life within a species
Longevity	The long duration of individual life
Multimorbidity	Coexistence of two or more medically (somatic or psychiatric) diagnosed chronic (not fully curable) or long-lasting (at least 6 months) diseases, of which at least one is of a primarily somatic nature
Senescence	The endogenous process of accumulative biological changes in the passage of time resulting in functional deterioration (Note: in biogerontology, senescence describes only one of the hallmarks of aging)

As discussed, it is currently challenging to measure biological age in the clinical setting. Therefore, we here discuss several approaches — comprehensive geriatric assessment (CGA), CGA-related instruments, CGA-based multidimensional prognosis — for their value in estimating aspects of age in an older individual beyond chronological age.

## Can CGA measure intrinsic aging?

CGA is an established approach for the evaluation of multifactorial aspects of age and aging falling outside the range of factors commonly used for clinical decision making, including multimorbidity, chronological age, and organ-related measures. The latter, in fact, show huge limitations in adequately illustrating recovery potential, outcomes, and trajectories of health in advanced age, as shown for example during the current SARS-CoV-2 pandemic [24].

CGA as a multidimensional, multidisciplinary process which identifies medical, social, and functional needs, accompanied by the development of an integrated/coordinated care plan to meet those needs [25], has been shown to be an effective approach to care of older people in a range of different healthcare settings (hospital, long-term care, nursing homes, primary care) and clinical conditions including orthogeriatrics, surgical and medical care, cancer, and dementia [26–29]. In line with the above mentioned “terminology issue” affecting the field of aging medicine, there is still a fundamental need for harmonization of instruments aimed at identifying older persons benefitting from multidisciplinary geriatric interventions. Multidimensional screening tools — suitable for a generic evaluation preceding targeted diagnostics — are in fact too often being performed with expectations of successful clinical characterization and tailored interventions. The latter, however, are per definition tasks of a CGA, which needs specialized skills in order to be performed without unnecessary time cost [30, 31]. These skills are irreplaceable even for the correct performance of compact screening versions. Compact screening tools would be able to direct a more comprehensive assessment to specific conditions to persons most likely to need it, with the ultimate scope of promoting a parsimonious distribution of healthcare resources. A challenge to the wider utilization of the CGA [20] is the need for gerontological training, as clinical trials that evaluated the effect of CGA without involving geriatricians have not been shown to be effective [32, 33].

Several tools for CGA have been developed in different countries, some of which became a harmonic suite of assessment instruments. The “international Resident Assessment Instrument” (interRAI, www.interrai.org), for instance, arises from a so-called *Minimum Data Set*, a suite of assessments which are reliable, valid, compatible with electronic health records and updated with emerging progress in gerontology and health sciences. The “MAnageable GeriatrIC assessment — MAGIC,” developed in Germany, is especially tailored to the requirements of daily primary care [34]. These and many other CGAs across countries and languages worldwide, if adopted in the validated correct way, not only allow seamless transfer of information between care settings but also can raise alerts for further in-depth assessments. Although several CGAs have been validated in many clinical settings (hospital, long-term care, ambulatory, community, and population level) and clinical conditions [27, 35], a caveat to their use is that their findings actually trigger appropriate care intervention so as to avoid the risk of assessment without action [36].

Despite the challenges associated to the correct and therefore successful performance of the CGA, there is no doubt that CGA-based tools can measure overall health and functional status in advanced age. The methodologies currently developed can track these through adult life. A challenge remains as to whether it is possible to disentangle age-related morbidity and disability from the processes of aging which are considered to relate to intrinsic biological aging. As discussed, frailty, currently considered as a multidimensional condition beyond chronological age informing at least in part on biological age [37], can be well measured by the CGA [38, 39]. However, it is essential to evaluate frailty as an independent entity, separated from specific diseases or other conditions.

In agreement with the aphorism of the celebrated pioneer of geriatric medicine, Bernard Isaacs, that “if you design for the old, you shall include the young,” the interRAI has proven to be effective in screening for functional and psychosocial problems in patients admitted to hospital from the age of 18 upwards, with at least one geriatric syndrome detected in 34.6% of those aged under 50 and 38.9% in those aged 50–69 years [38]. According to the multidimensional nature of frailty beyond chronological age and organ reserve reduction, frailty indexes are being developed which derive from CGA tools [38, 40, 41], although several of them might need to be further evaluated and implemented.

No current processes of CGA include putative markers of biological aging [42], and an interesting debate could arise from conceptualizing whether it is possible to control for risk factors known to contribute to relevant age-related disease in future studies which include such markers. For established risk factors, it is worth noting that several geriatric syndromes, whose metabolic basis is largely accepted, display a vascular component [43]. The study of vascular dysfunction has generated many observations and translational findings. However, more challenging is the control of factors more recently associated with rate reduction of neurodegenerative disease, such as education as a protective factor for dementia [44, 45].

What has been missing until recently from approaches such as frailty assessment and CGA is a measure of resilience [46]. In an encouraging development, the interRAI Home Care (HC) CGA has been used to develop a Recovery Algorithm [47]. This measure predicts recovery, with improvement rates rising from 6.9 to 47.2% across the 7 levels of the algorithm. Of note, the measure includes an interRAI HC item which reflects whether the home care service recipient believes he or she is capable of increased functional independence: this psychological element has been associated with physical resilience [48].

In summary, if the operationalization of variables that capture multidomain aspects of biological aging — which eventually lead to loss of resiliency to internal and external stressors, and facilitate the commingling of disability and frailty — has proven complex, CGA represents the most promising platform upon which to develop future exploration of the subject. In the interim, the frailty outputs of CGA and CGA-based prognostic evaluation as described below allow for an approximation of current concepts of biological aging or physical fitness relative to aging cohorts across the lifespan. The longitudinal, dynamic nature of CGA tools allows for measurement of response, resilience, and decline to emergent stressors over time.

## Is multidimensional prognosis an indicator of dynamics of aging?

A solid knowledge of the multifactorial biomolecular basis of aging mediates the rationale for a comprehensive approach to the older person, especially the frail multimorbid older patient, in order to develop a goal-oriented and patient-centered clinical management of the patient. Due to its efficacy in exploring multiple domains of health, the CGA determines clinical profile, disease risk, and intrinsic capacity to shape a “personalized” therapeutic and care plan to the older patient. In this context, a shared clinical decision making based on information on prognosis, i.e., life expectancy and quality of life, is a key point for contemporary medicine.

Among numerous recently developed tools to predict death — a crucial element in medicine [49] —, the multidimensional prognostic index (MPI) is the only one based on CGA. The MPI uses a mathematic algorithm including information about eight domains relevant for the global assessment of the older person (functional and cognitive status, nutrition, mobility and risk of pressure sores, multi-morbidity, polypharmacy, and co-habitation), to generate a numeric score (or index) ranging between 0 and 1 and expressing the global risk of multidimensional impairment. Initially developed and validated as a prognostic index predicting mortality in hospitalized older patients [50], a series of multicenter studies demonstrated that the MPI is able to (1) predict mortality more accurately than other frailty instruments based on both phenotypic and multiple-deficits models [51]; (2) predict in-hospital length of stay [52, 53]; (3) monitor changes of health and functional status during hospitalization [54, 55]; (4) identify those older patients who will be admitted to homecare services, nursing homes and/or re-hospitalized one-year after discharge from the hospital [56]; (5) inform about health-related quality of life in older patients admitted to emergency department [57]; (6) predict burden on healthcare resources [58] and successful application for disability social benefits in older people with cognitive decline [59]. Finally, systematic reviews reported that MPI was a CGA-based prognostic tool with good discrimination, accuracy, and calibration [60], useful in both clinical practice and research [61], and showing a very high validity, reliability, and feasibility compared to other tools used to identify frail older patients [62].

More recently, other versions of the MPI have been developed and validated in community-dwelling older subjects worldwide who underwent a CGA, confirming the accuracy of the MPI in predicting life expectancy, the risk of hospitalization during long periods of follow-up (up to 15 years), as well as risk of incident depression, falls, and cardiovascular diseases [63–70]. The MPI, even in its self- and telemedically administered versions, is able to express numerically global health and functions enabling a multidimensional approach to frailty management [24]. Indeed, the MPI is currently one of the most commonly used tools for evaluating frailty, in both primary care and hospital settings [39].

The approach by means of the MPI, as currently single available CGA-based prognostic index able to capture the dynamic, multidimensional features of poor outcome occurrence, not only represents a first attempt to move out from Plato’s cave — by acting as an indicator of aging rate changes —, but it paves the way to a better clinical decision making (i.e., to treat or not to treat) still largely depending on physicians’ attitudes towards older patients. Accordingly, and as displayed in Table 2, several clinical studies evaluated the appropriateness of “critical” treatments in older multimorbid patients, disclosing recommendations of great potential interest [65, 71–94]. Clearly, the MPI is unable to fully capture the complexity of the multiple aspects of biological aging. However, it represents a measure of the dynamic functional state of the person, which can provide guidance while planning interventions.

**Table 2:** Examples of potential of the multidimensional prognostic index (MPI) for clinical decision making in older subjects with specific clinical conditions

Clinical conditions [references]	Type of study and sample size	Outcome	Main message
Metabolic disorders			
Malnutrition and dysphagia [71]	Observational longitudinal multicenter study of 1064 patients ≥ 65 years treated vs not-treated with enteral tube feeding (ETF)	1-year mortality	ETF is associated with higher risk of death only in more frail patients (MPI-3)
Diabetes mellitus (DM) [72, 73]	Retrospective studies of 1342 [72] and 1712 community-dweller patients ≥ 65 years with DM, treated vs not-treated with statins [73]	Hypoglycemic events, hospitalization for glycemic decompensation	The MPI may identify patients at highest risk for hypoglycemic events and hospitalization for glycemic decompensation
Cardiology			
Coronary artery disease (CAD) [74]	Retrospective study of 2597 community-dwellers patients ≥ 65 years with CAD treated vs not-treated with statins	3-year mortality	Statin use was associated with lower mortality independently of age and frailty grade (MPI)
Acute myocardial infarction (AMI) [75]	Observational longitudinal of 241 patients ≥ 65 years undergoing percutaneous coronary intervention	1- and 6-month mortality, length of hospital stay (LOS), hospital complications	MPI-3 patients had higher risk of 1- and 6-month mortality, greater LOS and in-hospital complications
Atrial fibrillation (AF) [76]	Retrospective study of 1827 community-dwellers patients ≥ 65 years with AF treated vs not-treated with anticoagulants	2-year mortality	Older adults with AF benefitted from anticoagulation in terms of lower all-cause mean 2-year mortality regardless of MPI grade
Transcatheter aortic valve implantation (TAVI) in aortic valve stenosis [77–79]	Observational prospective studies of 116 patients ≥ 75 years [77], 71 patients ≥ 80 years [78], and 376 patients [79] who underwent TAVI	6- and 12-month mortality [77], and/or non-fatal stroke [78]; 1-, 2-, and 3-year mortality [79]	MPI-3 patients had higher 6- and 12-month mortality, 3-year mortality, re-hospitalization, and/or non-fatal stroke
Percutaneous repair of tricuspid and mitral valves [80]	Observational prospective study of 226 patients undergoing transcatheter tricuspid and mitral valve repair	Procedural outcomes and 6-month mortality	MPI was associated with 6-month mortality, not with procedural efficacy and safety
Heart failure [81]	Observational prospective study of 365 patients ≥ 65 years with diagnosis of heart failure	1-month mortality	Increasing MPI grade associated with higher rates of 1-month mortality
Pulmonary/infectious diseases			
Community-acquired pneumonia (CAP) [82–84]	Observational prospective studies of 50 patients [82], 49 patients [84], and 134 patients [83] ≥ 65 years hospitalized with CAP	1-month mortality [82, 84] and 1-, 6-, and 12-month mortality [83]	MPI predicted 1-month mortality. Proadrenomedullin [82] and procalcitonin [84] improved prognostic accuracy of MPI. Higher MPI was associated with 1-,6-, and 12-month mortality [83]
Acute respiratory failure [85]	Retrospective observational study of 231 older patients receiving non-invasive ventilation (NIV) vs not-NIV	In-hospital mortality	Higher MPI at admission predicted in-hospital mortality
SARS-CoV-2 infection [86, 87]	Multicenter observational prospective studies of 227 patients ≥ 65 years [86] and retrospective observational study of 100 patients ≥ 75 years [87]	In-hospital mortality and admission to intensive care unit (ICU) [86]; in-hospital, 1- and 3-month mortality [87]	MPI-3 patients had higher in-hospital mortality and longer LOS. No effect on admission to ICU [86]. Frailty, identified by MPI, was associated with mortality [87]
SARS-CoV-2 infection in nursing homes (NH) [88]	Retrospective propensity score-adjusted study of 3946 older NH residents with or without COVID-19	Mortality	Increasing MPI associated with higher rates of mortality
Oncology			
Colorectal cancer [89]	Observational longitudinal of 104 older patients receiving surgery	90-day postoperative complications	MPI was associated with major postoperative complications
Advanced cancer [90]	Observational longitudinal of 79 older patients receiving immunotherapy	Rate of survival	MPI predicted rate of survival
Nephrology			
Chronic kidney disease (CKD) III-V [91] and CKD [92]	Observational longitudinal studies of 173 patients ≥ 65 years receiving dialysis or conservative therapy [91], and 1198 patients ≥ 65 years with a diagnosis of CKD [92]	Hospitalization and 24-month survival	MPI was associated with days of hospitalization and rate of survival [91]. Adding MPI to eGFR predicted mortality more accurately [92]
Cognitive disorders			
Dementia [93]	Observational retrospective study of 6818 older community-dwellers dementia patients treated with anti-cholinesterasics or memantine VS no treatment	2-year mortality	Antidementia treatment was associated with reduced mortality in the MPI-1 and MPI-2 groups, but not in the MPI-3 group
Depression [46, 65, 94]	Longitudinal study of 1854 adults ≥ 65 years without depressive symptoms at baseline [65]; prospective study of 81 adults ≥ 60 years who underwent orthopedic surgery of the lower limb [46, 94] 485 adults ≥ 65 years with late-life major depressive disorder (MDD)	Development of depressive symptoms at 2-year follow-up [65]; MPI, resilience scale (RS), and functional independence measure (FIM) after a rehabilitation period [46]; response to treatment with selective serotonin reuptake Inhibitors (SSRIs) at 6-month follow-up [94]	Baseline MPI was associated with incident depressive symptoms [65]; disability at follow-up was negatively correlated with RS with interaction with MPI [46]; a baseline MPI < 0.1 was predictive of positive SSRIs treatment response [94]

While more research is needed to keep developing multidimensional prognostic indexes, actions are required which facilitate their calculation directly from information included in the hospital clinical records [95], the use of artificial intelligence and, of course, the further integration of biological markers [96, 97].

## Concluding remarks

We reviewed a set of tools to assess the rate of biological aging, their reliability, and indication for clinical utilization. CGA is an approved and widely used tool to measure functional capabilities, and its results can be compared as deviation from the age- and sex-specific reference performance assessed in representative healthy populations. Progress in the refinement of these tools is needed; in particular, their sensitivity and specificity in predicting multiple, geriatric-relevant health outcomes should be improved and they should become flexible enough to capture the essential variables in all particular persons. Estimating prognosis with the MPI, based on CGA, shows promise since it introduces a reliable measure of prognosis, and is therefore valuable for decision making and for establishing priorities in the allocation of resources. Some limitations of MPI may occur as the index depends on the CGA. Finally, it is important to highlight that although the MPI shows relevance in clinical practice, each clinician should strive to tailor the assessment tools to each specific patient to the best of his/her knowledge, with the goal of providing a precise and personalized care. It is also fundamental to consider how the patient’s perception of the condition of aging, health, or disease very often does not coincide with the clinician’s, and to bring the patient’s individuality to the center of the decision-making process.
